# Approaches in Physical Activity: From Basic to Applied Research 2017

**DOI:** 10.1155/2018/9732315

**Published:** 2018-12-04

**Authors:** Julien S. Baker, Bruce Davies, Leonardo dos Santos, Emmanuel Ciolac, Danilo Sales Bocalini

**Affiliations:** ^1^School of Health and Life Sciences, University of the West of Scotland, Hamilton Campus, Hamilton, Scotland, UK; ^2^Health and Exercise Science Research Laboratory, School of Applied Science, University of Glamorgan, Pontypridd, Wales, UK; ^3^Department of Physiological Sciences, Federal University of Espirito Santo, Vitoria, ES, Brazil; ^4^Exercise and Chronic Disease Research Laboratory, Department of Physical Education, São Paulo State University, Bauru, SP, Brazil; ^5^Experimental Physiology and Biochemistry Laboratory. Physical Education and Sport Center of Federal University of Espirito Santo, Vitoria, ES, Brazil

## 1. Introduction

Changes in the modern lifestyle including diets high in salt, sugar, and fat and low physical activity have contributed to the increasing incidence and prevalence of chronic diseases. Several nonpharmacological strategies have been developed aiming at promoting a healthy lifestyle to reduce drug dose and polypharmacy and decrease morbidity and mortality. This reform in the lifestyle is an attitude that should be encouraged in all sectors of health care.

In this way, special attention had been addressed to oxidative stress and free radical (FR). A FR is a molecule with an unpaired electron in its outer orbital and is produced during normal cellular metabolism [1]. High levels of radicals can damage cells by reacting with cellular components (e.g., proteins and lipids). This form of damage is called oxidation and can result in a lethal injury to all cells [2]. The adverse effects of excess free-radical formation have been hypothesized to lead to cancer, atherosclerosis, aging, and even exercise-associated oxidative damage. Aerobic organisms would not survive without mechanisms that counteract the detrimental effects of free radicals. The system includes the fat-soluble antioxidants such as vitamin E and beta-carotene (a vitamin A precursor); the major water-soluble antioxidant, vitamin C; antioxidant enzymes such as superoxide dismutase (SOD), catalase (CAT), and selenium-dependent glutathione peroxidase (GPX); and low-molecular-weight compounds such as glutathione [3]. These components preserve homeostasis during most normal cell function and mild oxidative stress. When free-radical production is excessive, however, or when the antioxidant system is overwhelmed, such as during nutritional deficiencies or exhaustive exercise, such imbalances may favour an “oxidative stress situation”

In the past two decades, accumulating evidence has shown that unaccustomed and strenuous exercise induces an imbalance between free-radical production and the body's antioxidant defence systems [4]. However, it remains unknown whether increased free-radical production is an unwanted consequence of exercise that promotes further inflammation and tissue damage, or if the body regulates oxidant production to control inflammation and repair. Performance also decreases in rats fed a vitamin E-deficient diet, thus implicating vitamin E in protecting against exercise-induced free-radical generation and injury (Zerba E et al., 1990).

Animal studies have shown promising results [5]: vitamin E supplementation at supraphysiologic doses for a minimum of 5 weeks can decrease lipid peroxide levels with exhaustive exercise. To be effective at all, vitamin E must be given for at least 2 weeks before exercise, and five times the RDA for vitamin E may be necessary to prevent free-radical damage. These findings should be balanced against human studies that have not demonstrated convincing evidence for exercise-induced oxidative stress damage. Duthie et al. (1990) found no difference in levels of plasma alpha- or beta-tocopherol in runners following a half marathon. Lovlin et al. [6] actually noted a decrease in plasma malondialdehyde levels, a marker for lipid peroxidation, in cyclists exercising at 40% to 70% VO_2_max. The selected articles produced here in this special addition present data looking at the mechanisms involved in oxidative stress.

The studies contained in this special issue include recent and topical research studies that investigate training adaptations for different populations, the effects of being physically active on lifestyle and different physiological systems, and the consequences of training. This exciting edition provides the reader with novel research investigating the role of physical activity on different outcomes and provides the researcher, scientist, and student with an interesting insight into the several adaptive process of on populations.
